# 

**Published:** 1895-09

**Authors:** 


NOTICE.

***All articles for publication, books for review, business communications, etc., must be sent to Editor, 1231 Locust Street, Philadelphia.***

***Subscribers will please notice that this Journal will be sent until arrears are paid and it is ordered discontinued.***

---

PHILADELPHIA:

No. 1231 LOCUST STREET.

